# Study on protective performance and gradient optimization of helmet foam liner under bullet impact

**DOI:** 10.1038/s41598-022-20533-9

**Published:** 2022-09-26

**Authors:** Xingyuan Huang, Qiujie Zheng, Lijun Chang, Zhihua Cai

**Affiliations:** Hunan University of Science and Technology, Xiangtan, 411201 China

**Keywords:** Mechanical properties, Mechanical engineering

## Abstract

Protective equipment in war plays a vital role in the safety of soldiers, the threat to soldiers from brain damage caused by deformation at the back of the helmet cannot be ignored, so research on reduce blunt post-cranial injury has great significance and value. This study first conducted gunshot experiments, used rifle bullets impact bulletproof plate and different density liner foam to record the incident process and internal response of craniocerebral model. After verifying the accuracy of finite element model through experimental data, optimization model is established based on response surface method to optimize the structure of gradient foam, analyze the cranial strain and energy absorption to select the best density and thickness distribution of each foam layer. Optimization results show that liner foam which designed to have lower density and thicker thickness for impact and brace layers, higher density and thinner thickness for middle layer can significantly improve the energy absorption efficiency. Compared to the 40.65 J of energy absorption before optimization, the optimized gradient foam can absorb 109.3 J of energy, with a 169% increase in the absorption ratio. The skull strain in the craniocerebral model was reduced from 1.260 × 10^–2^ to 1.034 × 10^–2^, with a reduction of about 22%. This study provides references for the design and development of protective equipment and plays an important role in ensuring the safety of soldiers in the battlefield environment.

## Introduction

In modern warfare, when kinetic energy or shock wave of bullets and fragments impacts the bulletproof helmet, the kinetic energy will be transmitted from helmet to skull and brain tissue, causing the rapid deformation, stretching, shearing, and final destruction of intracranial soft tissue, resulting in secondary brain damage^1^. Scholars have researched wound ballistics and biomechanics through experiments and numerical simulations^2–5^, Tham et al.^6^ conducted ballistic tests to study the response of KEVLAR helmet under bullet impact and found that KEVLAR helmet can withstand the impact of all-metal armored bullet traveling at speed of 358 m/s without being penetrated, and the helmet's V50 ballistic limit is 610 m/s. In early experimental studies, the main target was live anesthetized animals (such as pigs, dogs, and sheep). Rafaels et al.^7^ conducted statistical analysis on the brain damage caused by bullet impact bulletproof helmets through cadavers brain injury experiment. Due to moral restrictions and legal prohibitions in most countries, human tissue simulants (such as gelatin, soap, etc.) have been introduced into experiments in recent years. Gelatin is used in wound ballistic research because its mechanical properties are considered to be similar to those of human tissues^8–10^. Freitas et al.^11^ used the outer skull and inner soft tissue to study the dynamic response of the brain caused by the bullet impact of the bulletproof helmet. This experimental model can be used to intuitively measure the physical quantity of the brain injury. With the advancement of research related to the energy-absorbing properties of foam materials, foam was widely used as cushion material for helmets^12^. Tan et al^13^ conducted frontal (205 m/s) and lateral (220 m/s) impacts with 11.9 g spherical steel projectiles on two ACH helmets which without foam cushion structure and with Oregon Aero foam padding respectively. The results shows that for ballistic impacts, foam helps to reduce the impact force and provided better protection compared to the helmet without foam liner, and the softer and less rigid foam is more effective in shock absorption. Li et al.^14^ investigated the effect of foam cushion stiffness on brain injury risk. Salimi et al.^15^ studied the improvement of protective performance of EPP and EPS foam helmet linings with different densities. Among helmet cushioning materials, functionally graded materials have attracted extensive attention due to superior performance over homogeneous materials^16–19^, and designers can obtain the best energy absorption effect by adjusting the gradient distribution. Zhang et al.^16^ simulated the energy absorption of functionally graded foams under the medium and high speed impact of spheres by numerical simulation method. Koohbor et al. ^18^ proposed an optimal design of graded foams by investigating the effect of density gradation on the load-bearing and energy-absorbing properties of graded foams, and studied the intrinsic response and energy absorption of continuous gradient and discrete laminar foams. Liu et al.^19^ theoretically investigated the impact resistance and energy absorption capacity of foam rods with nonlinear gradient (positive and negative gradients) distribution under mass projectile impact, analyzed the response of foam rods at the impact and brace end, and performed related simulation analysis. In addition, functional gradient foams can be used to optimize the graded structure by adjusting the layering ratio to obtain the desired mechanical properties^20^. What’s more, researches on lightweight materials also pointed out that 3D printed bio-inspired porous structure through additive manufacturing can improve the energy absorption effect of armor^21–23^.

Existing studies mainly focused on the density of helmet shell and liner foam, rarely considers the effect of each layer foam thickness on protective performance. Because the response of "protective equipment- foam liner-cranial brain" as a whole to bullet impact and the interaction is complex, analyzing the mechanical properties of the material itself for the protective liner is insufficient. In order to conduct a more in-depth investigation of the gradient foam liner, this study performs high-speed bullet impact experiments and analyzes the dynamic response of the cranial physics model. Then the parameters of functional gradient foam liner with variable thickness and density are optimized based on Response surface method (RSM) and finite element model, the optimized parameters are fed back to finite element model for verification and further investigation of gradient foam liner optimal performance, which provides references for the design of protective equipment such as bulletproof helmets and bulletproof panels, and provides protection for the safety of soldiers on the battlefield.

## Materials and methods

### Rifle impact experiment

**Figure 1:**
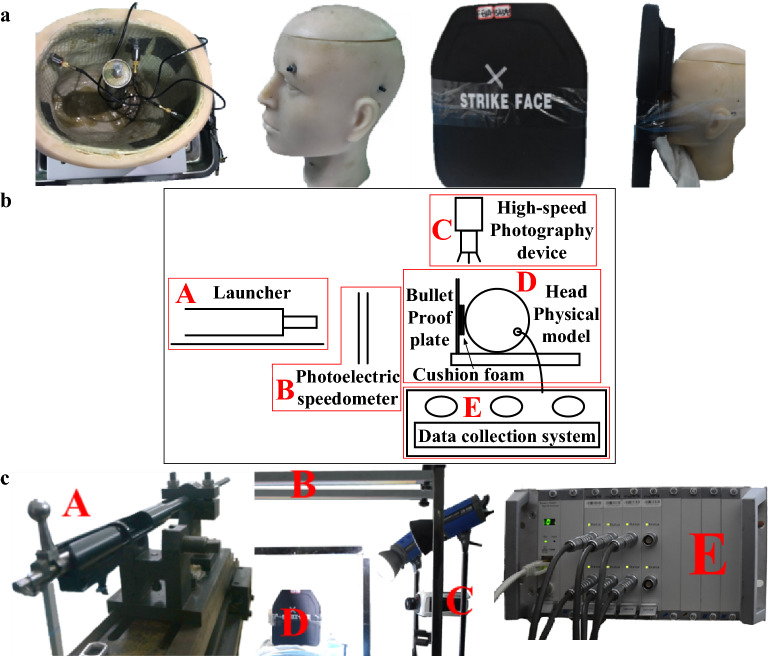
Rifle impact experiment preparation. (**a)** Head model assembly process (Images were processed by Microsoft Paint). (**b)** Schematic diagram of bullet impact experiment (Image was processed by Microsoft Office Visio 2016). (**c)** Layout of bullet impact experiment (Images were processed by Microsoft Office PowerPoint 2010).

The U.S. Enhanced Combat Helmets (ECH) recently proved to be able to withstand rifle bullets at high speeds^24^. Using the same material as ECH^25^, bulletproof plate made of ultra-high molecular weight polyethylene was chosen for this experiment. By comparison with helmets, bulletproof plates are more convenient for observing the dynamic response, avoiding penetrating damage to the head model, allowing the model to be reused, and reasonably controlling the costs of experiments. Conjunctival-fibrious polyurethane elastic material is used to create the skin of the head, high phosphorus and high calcium thermosetting resin is used to create the skull, and gelatin is used to make the brain tissue^26^. Firstly, drill holes on the skull of model to fix and install the acceleration sensors (KD1000D, KD1001A, KeDong Electronic Inc.) and pressure sensors (KD2004G02X, KeDong Electronic Inc.) inside model with the sensitive surface facing impact direction. Upon installation, the sensor is connected to multi-channel dynamic signal acquisition system and tested to ensure normal use, the rifle bullet launching device is facing the shooting aim point marked on bulletproof plate. Attach the model, bulletproof plate, and cushion foam to the experimental bench. Density configuration of each foam layer is shown in Table 1, the first foam layer is the impact layer, the second layer is the middle layer, and the third layer is the brace layer, which is contact with the head directly, the size of each foam layer is 100 mm × 100 mm × 10 mm, assembly process is shown in Fig. 1a–c shows the experimental site and equipment set up with the schematic diagram of rifle bullet impact. Bullet speed is measured by photoelectric device between the launching device and experimental platform, high-speed photograph device on the side of the platform is used to capture the process of bullets impact the bulletproof plates. After the installation was completed, 5.56 mm rifle bullets were fired to conduct frontal impact experiments on the head model.

**Table 1:** Density configuration of each foam layer.

	Impact layer (kg/m^3^)	Middle layer (kg/m^3^)	Brace layer (kg/m^3^)
Homogeneous 30 (H30)	30	30	30
Homogeneous 45 (H45)	45	45	45
Homogeneous 60 (H60)	60	60	60
Positive density gradient (POS)	30	45	60
Negative density gradient (NEG)	60	45	30
Convex density gradient (CVX)	30	60	30
Concave density gradient (CVE)	60	30	60

### Finite element model

#### Head finite element model

**Figure 2:**
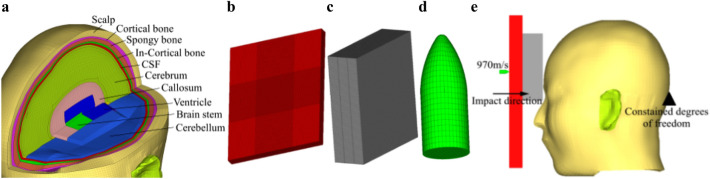
Finite element model. (**a)** Head finite element model (Image was processed by Microsoft Office PowerPoint 2010). (**b)** Bulletproof plate finite element model. (**c)** Cushion foam finite element model. (**d)** Bullet finite element model. (**e)** Impact model (Image was processed by Microsoft Office PowerPoint 2010).

The head finite element model has been established in the early stage, includes scalp, bone tissue (cortical bone, spongy bone, in-cortical bone, face bone, mandible), soft tissue (csf, cerebrum, callosum, ventricle, brain stem, cerebellum), membrane tissue (pia mater, dura mater, tentorium cerebelli, falx cerebri), as shown in Fig. 2a. The brain pressures, skull responses and the brain skull relative displacements have been verified refer to the relevant literature^27–29^, the material properties of bones and brain tissue are shown in Tables 2 and 3. It can be used to study the dynamic response of the brain under the impact of rifle bullets and conduct injury analysis.

**Table 2:** Material properties of bones^27–29^.

	Density (g/cm^3^)	Young’s modulus (GPa)	Poisson’s ratio	Shear modulus (GPa)	Hardening parameter	Plastic failure strain (%)
Cortical bone	2.0	11.5	0.3	1.15	0.1	0.02
Mandible	2.0	11.5	0.3	1.15		0.02
Face bone	5.0	21	0.23	1.15		0.02
Spongy bone	1.0	0.04	0.45	0.001		0.03

**Table 3:** Material parameters of brain tissue^27–29^.

	Density (g/cm^3^)	Young’s modulus (MPa)	Poisson’s ratio	Shear modulus(kPa) $G(t) = G_{0} + (G_{0} - G_{\infty } )e^{ - \beta t}$			Bulk modulus (MPa)
				$G_{0}$	$G_{\infty }$	$\beta$	
Scalp	1.0	16.7	0.42				
CSF	1.05			100	20	100	4.97
Callosum, ventricle, falx cerebri, tentorium cerebelli	1.14	31.5	0.45				
Brain stem, cerebellum, cerebrum	1.04			1.66	0.928	16.95	557
Pia mater	1.13	31.5	0.23				
Dura mater	1.13	11.5	0.45				

#### Bulletproof plate finite element model

The bulletproof plate finite element model is established in HyperMesh (Altair Engineering Inc., Troy, MI, USA) size of bulletproof plate is 350 mm × 200 mm × 20 mm. Within the same deviation range, considering the calculation efficiency, the grid of bullet impact area is encrypted to 1 mm × 1 mm × 1 mm, remaining grid size is 2 mm × 1 mm × 1 mm, and the number of grids is 680,000, as shown in Fig. 2b. The material parameters of the ultra-high molecular weight polyethylene fiber are described in Table 4, which is simulated using MAT22 (MAT_COMPOSITE_DAMAGE)^30,31^.

**Table 4:** Material parameter of bulletproof plate^30,31^.

$\rho$(kg/m^3^)	*E*_*a*_ (GPa)	*E*_*b*_ (GPa)	*E*_*c*_ (GPa)	*PR*_*ba*_
970	1.97	30.7	30.7	0.008
*PR*_*ca*_	*PR*_*cb*_	*G*_*ab*_/GPa	*G*_*bc*_/GPa	*G*_*ca*_/GPa
0.044	0.044	0.67	1.97	0.67
*SYZ*/GPa	*SZX*/GPa	*SC*/GPa	*XT*/GPa	*YT*/GPa
0.95	0.95	0.36	3.0	3.0
*SN*/GPa	*YC*/GPa	*KFAIL*/GPa	*AOPT*	*ALPH*
0.95	2.5	2.2	0	0.5

*Ea*, *Eb*, *Ec* Young’s modulus, *PRba*, *PRca*, *PRcb* Poisson’s ratio, *Gab*, *Gbc*, *Gca* shear modulus, *SYZ*, *SZX* transverse shear strength, *SC* shear strength, *XT* longitudinal tensile strength, *YT* transverse tensile strength, *SN* normal tensile strength, *YC* transverse compressive strength, *KFAIL* bulk modulus of failed material, *ALPH* nonlinear term shear stress.

#### Cushion foam finite element model

The cushion foam finite model is established in Hypermesh, the size of the foam is 100 mm × 100 mm × 30 mm, as shown in Fig. 2c, the grid size is 1 mm × 1 mm × 1 mm, and the number of grids is 300,000. Choose the same foam material as the impact test, simulate its material properties with MAT57 (MAT_LOW_DENSITY_FOAM).

#### Bullet finite element model

The 5.56 mm bullet finite model is established and meshed in Hypermesh. As shown in Fig. 2d, the grid size is 0.5 mm × 0.5 mm × 0.5 mm, the number of grids is 2900. Copper's thermal viscoplastic response is simulated using Johnson–Cook in bullet model. The specific material parameters are shown in Table 5^32^.

**Table 5:** Rifle bullet material parameter^32^.

*A* (GPa)	*B* (GPa)	*C*	*n*	*m*
0.09	0.292	0.025	0.31	1.09

$\rho$(kg/m^3^)	Specific heat (J/kg·K)	Shear modulus (GPa)	Bulk modulus (GPa)	
8950	1.75	47.27	102.4	

#### Impact model

Combine bullet, bulletproof plate, cushion foam and head model, restrict the freedom of bulletproof plate in six directions (the translational and rotational degrees of freedom in XYZ directions) according to the experimental conditions. Due to the extremely short impact time, the impact of the head's own motion is not considered, so the six degrees of freedom of head are restrained in impact direction at the same time. In the experiment, the photoelectric velocity measurement system measured the velocity of the bullet was 970.78 m/s, so the rifle bullet model was given an initial velocity of 970 m/s, and the final rifle bullet impact model is shown in Fig. 2e.

### Response surface methodology optimization design scheme

**Figure 3:**
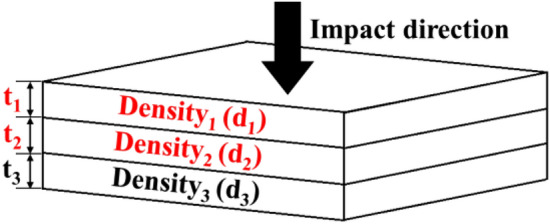
Schematic diagram of gradient foam (Image was processed by Microsoft Office PowerPoint 2010).

Optimized object is the overall structure of three-layer variable thickness and variable density foam, the design variables are the thickness and density of the first two foam layers near the impact end, with a total of four independent variables, as shown in Fig. 3. The overall thickness (*t*_*1*_ + *t*_*2*_ + *t*_*3*_ = 30 mm) and density of the foam (surface density = 1.35 kg/m^2^) are fixed values. In order to avoid extreme values which do not conform to actual situation at the design point, the four variables are constrained according to the actual situation, and the thickness and density of the brace layer (*t*_3_, *d*_3_) are determined accordingly to (*t*_1_, *d*_1_), (*t*_2_, *d*_2_). The objective function is the ratio of actual energy absorption value and the ideal energy absorption value, the functional gradient foam performance optimization problem is defined as shown in formula (1).

1 $$ \left\{ \begin{gathered} \min \, R_{E} = f(t_{1} ,t_{2} ,d_{1} ,d_{2} ) \hfill \\ s.t. \, 6mm \le t_{1} \hfill \\ \, t_{2} \le 14mm \hfill \\ \, 30kg{/}m^{3} \le d_{1} \hfill \\ \, d_{2} \le 60kg{/}m^{3} \hfill \\ \, 6mm \le 30 - t_{1} - t_{2} \le 14mm \hfill \\ \, 30kg{/}m^{3} \le (1.35 - t_{1} d_{1} - t_{2} d_{2} ){/}(30 - t_{1} - t_{2} ) \le 60kg{/}m^{3} \hfill \\ \end{gathered} \right. $$

*t*_1_ is the thickness of the impact layer foam, *t*_2_ is the thickness of the middle layer foam. *d*_1_ is the density of the impact layer foam, *d*_2_ is the density of the middle layer foam. *R*_E_ is the objective function values about *t*_1_, *d*_1_, *t*_2_, *d*_2_.

For the objective values of single design point, calculated as formula (2).

2 $$ R_{Ei} = E_{Ai} /E_{I} = E_{Ai} /(1 + 20\% )E_{A\max } $$

*E*_*Ai*_ is the actual energy absorption value of the $i$th simulation, *E*_*Amax*_ is the maximum value of actual energy absorption in all simulations. *E*_*I*_ is the ideal energy absorption value, which improves the energy absorption performance by 20% compared to the maximum actual energy absorption value.

**Figure 4:**
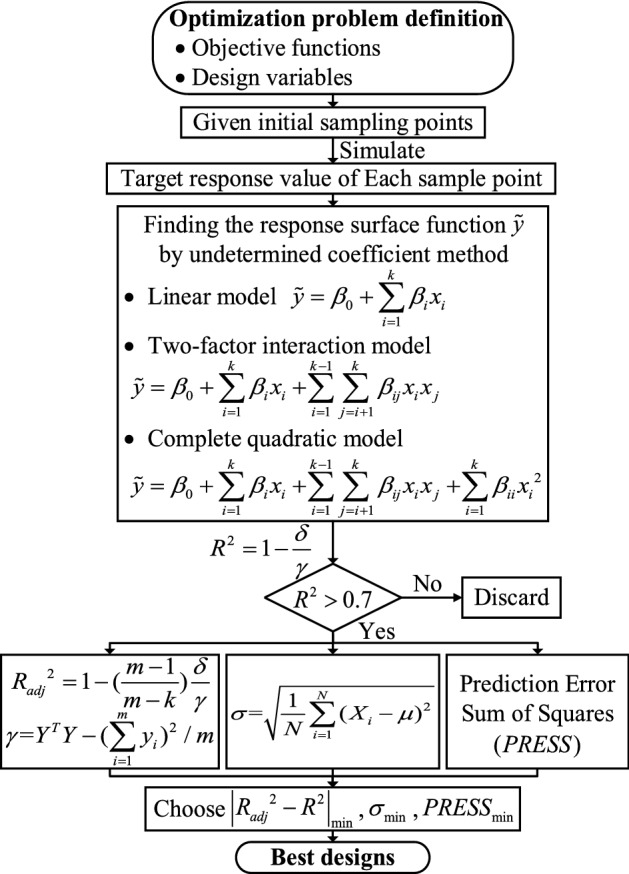
Flow chart for response surface methodology optimization.

After determining the design variables and objective function, exact functional relationship between independent variables and response values is obtained using the RSM, the main process is shown in Fig. 4.

## Result and discussion

### Bullet impact experiment results

**Figure 5:**
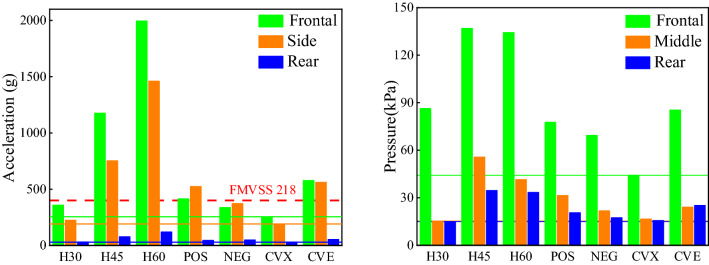
Peak cranial acceleration and intracranial pressure for each impact experiments.

The peak cranial acceleration and intracranial pressure of each foam combination collected from experiments are shown in Fig. 5. It can be seen from the acceleration results that the gradient structure has a substantial decrease in the overall acceleration compared with homogeneous structure. Considering the short acceleration peak action time in the experiment cannot be analyzed by the head injury criterion (HIC), the safety criterion of not exceeding 400 g peak acceleration proposed by the U.S. Federal Motor Vehicle Safety Standard 218^33^ (FMVSS 218) is used for comparative analysis. After adopting the gradient structure, it can significantly reduce the head acceleration value, under the high speed bullet impact of 970 m/s, the H30, NEG and CVX can still play good role in protecting the head. Combined with the intracranial pressure results, the CVX shows the strongest comprehensive performance, and can reduce the overall weight of the foam structure^34^.

### Finite element model verification based on impact experiment

**Figure 6:**
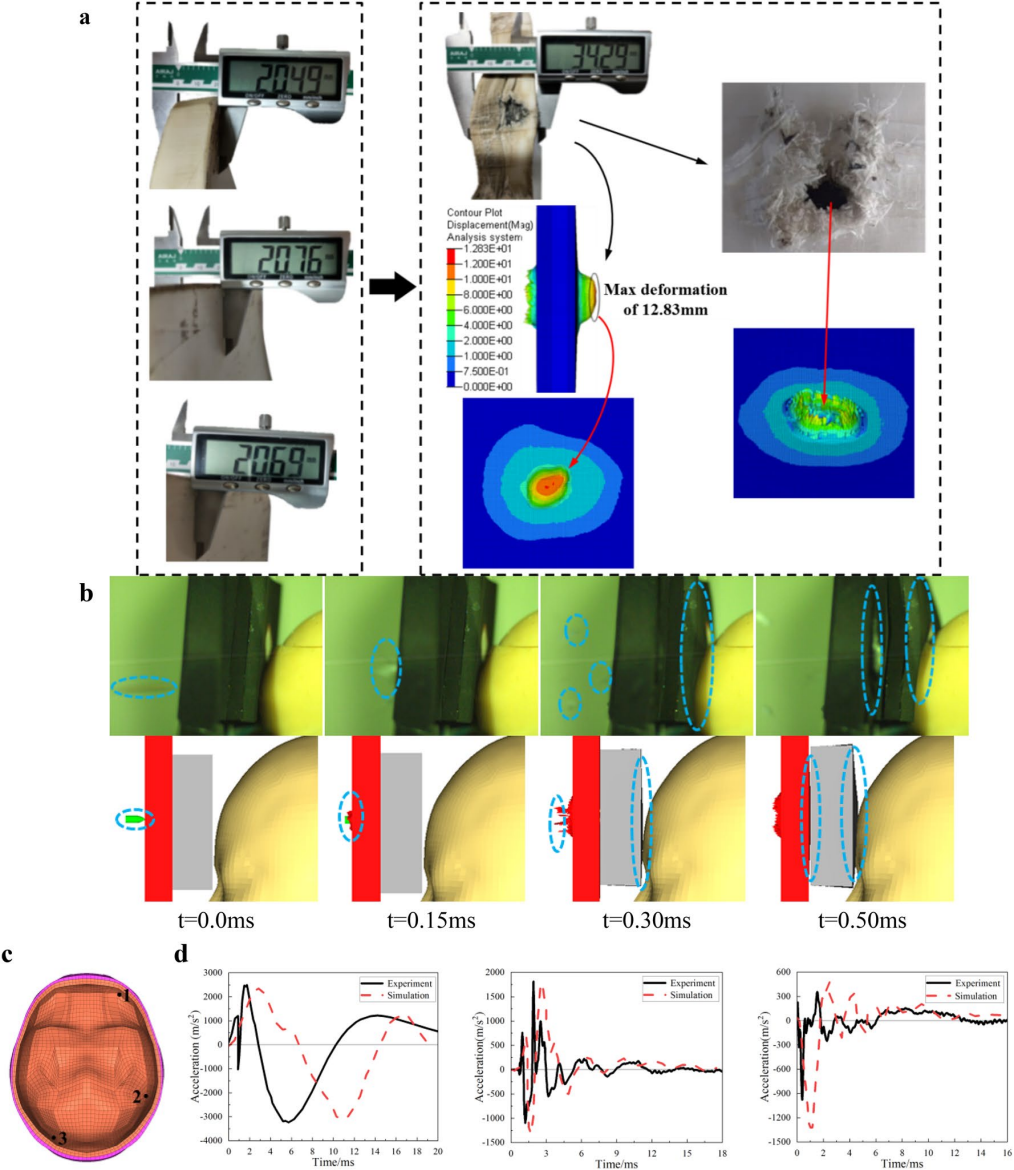
Finite element model verification based on bullet impact experiment. (**a)** Comparisons of deformation and fracture pattern of the bulletproof plate. (**b)** Comparisons of bullet impact process between experiment and simulation. (**c)** Schematic diagram of the points location in head finite element model. (**d)** Skull acceleration of each selected point.

Choose the CVX experiment data to verify simulation results, the average initial thickness of bulletproof plate is taken as (20.49 + 20.76 + 20.69)/3 = 20.64 mm. After the impact experiment, the size of deformation area, the profile of maximum deformation, the ruptures of bulletproof plate at the incident point and the corresponding damage of bulletproof plate in the simulation are shown in Fig. 6a. In simulation, the maximum deformation of bulletproof plate is 12.83 mm, while the maximum deformation in experiment is 13.65 mm (34.29 mm-20.64 mm). The convex deformation area of bulletproof plate is 75 mm × 70 mm in experiment, and 82 mm × 66 mm in simulation.

The comparisons between experiment and simulation are shown in Fig. 6b. Refer to the installation position of acceleration sensor in physical head model (Fig. 1a), select the same position in finite element model (Fig. 6c) to output the skull acceleration value and compare it with the sensor data (Fig. 6d). The initial time of the experimental curve is the moment when corresponding sensor begins recording effective data (non-zero data). There are some differences between the experiment results and the simulation results, the phase difference between the acceleration curve measured by the sensor and the simulated curve at point 1 is larger than that at point 2 and 3, because the sensor at point 1 is directly impacted by the high-speed bullet in the experiment, which causes it to reach the peak acceleration in a short period of time, while the sensors in the middle and posterior parts are subjected to forces originating from the transmission of gelatin inside the skull. The point locations in the model deviates slightly from the experiment, as well as the material properties and parameters of the finite element model are not exactly the same as the physical head model, so there are delay in the acceleration change and different phase differences between the simulation results and experiment results. The oscillations in both the middle and posterior sensors and the simulation model measurement points are caused by the repeated action of stress waves in the brain, and the anterior sensor has a smoother curve because it is close to the liner foams, which absorbs some of the energy. But the overall peak value and the trend of change are consistent, which proves the effectiveness of finite element model.

**Figure 7:**
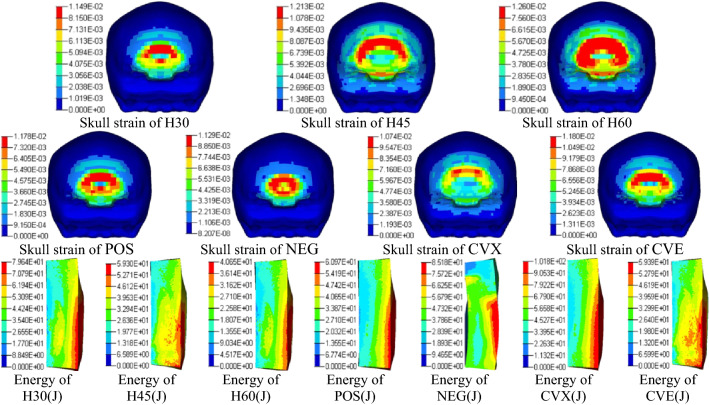
Cloud map of peak skull strain and foam energy absorption.

After the finite element model was validated, simulation results were further validated by outputting the peak skull strain and energy absorption of the liner foam**.** As shown in Fig. 7 (the order is H30, H45, H60, POS, NEG, CVX, CVE), the left side of foam is impact layer and the rightmost is brace layer. The foam absorbs energy transmitted by the shock wave through its own deformation, and brace layer produces the largest deformation due to direct contact with the head. It can be seen from the figure that the area of skull strain and large deformation area are relatively small and energy absorption are higher than other conditions when the foam combination are NEG and CVX. The best performance of CVX is consistent with the results obtained from the bullet impact experiment, which further verifies the accuracy of finite element model.

### Optimization analysis of gradient foam protection performance based on RSM

Verified finite element model is used for simulation, the energy absorption ratio of each design point is obtained by formula (2). Table 6 shows the parameter values designed by RSM which imported to DESIGN- EXPERT (StatEase Inc., Minneapolis, USA) for foam gradient structure optimization and analysis. The equations and solution accuracy of different response surface forms are shown in Table 7.

**Table 6:** Design points and corresponding simulation results.

No	t_1_ (mm)	t_2_ (mm)	d_1_ (kg/m^3^)	d_2_ (kg/m^3^)	R_E_
1	6	10	45	45	0.681738
2	10	10	45	30	0.67133
3	8	10	45	60	0.775002
4	10	10	30	60	0.602248
5	14	6	30	60	0.664202
6	14	6	45	45	0.645056
7	14	10	45	60	0.774658
8	10	14	45	45	0.556116
9	10	10	60	45	0.828296
10	10	6	30	45	0.589975
11	6	14	45	60	0.794099
12	6	14	60	30	0.738625
13	10	6	45	60	0.768998
14	10	14	60	45	0.833333
15	6	10	60	45	0.822395
16	8	10	30	45	0.693166
17	14	10	30	45	0.576426
18	8	14	60	45	0.813637
19	8	14	30	60	0.582218
20	10	10	30	45	0.670174
21	14	6	30	45	0.602542
22	10	6	60	30	0.60647
23	6	14	30	45	0.76537
24	10	10	60	30	0.758851
25	14	10	60	30	0.734427

**Table 7:** Equations and solution accuracy of different response surface forms.

	$R^{2}$	$R_{adj}^{2}$	$\sigma$	$PRESS$
**Linear model** $$\begin{gathered} R_{E} = f(t_{1} ,t_{2} ,d_{1} ,d_{2} ) = \beta_{0} + \sum\limits_{i = 1}^{k} {\beta_{i} x_{i} } \hfill \\ = {0}{\text{.338259}} - {0}{\text{.00571203}}t_{1} + {0}{\text{.00218015}}t_{2} \hfill \\ { + 0}{\text{.00538606}}d_{1} { + }0.0{0342933}d_{2} \hfill \\ \end{gathered}$$	0.5500	0.4600	0.0660	0.1392
**Two-factor interaction model** $$\begin{gathered} R_{E} = f(t_{1} ,t_{2} ,d_{1} ,d_{2} ) = \beta_{0} + \sum\limits_{i = 1}^{k} {\beta_{i} x_{i} + } \sum\limits_{i = 1}^{k - 1} {\sum\limits_{j = i + 1}^{k} {\beta_{ij} x_{i} x_{j} } } \hfill \\ = {0}{\text{.703209}} - 0.0{21504}t_{1} + {0}{\text{.0502362}}t_{2} \hfill \\ - 0.0{138276}d_{1} - 0.0{00391668}d_{2} - 0.00{202628}t_{1} t_{2} \hfill \\ + 0.00{0748343}t_{1} d_{1} - 0.000{0601363}t_{{1}} d_{2} \hfill \\ + 0.000{147248}t_{2} d_{1} - 0.00078242t_{2} d_{2} + 0.000238976d_{1} d_{2} \hfill \\ \end{gathered}$$	0.7140	0.5097	0.0629	0.1730
**Complete quadratic model** $$\begin{gathered} R_{E} = f(t_{1} ,t_{2} ,d_{1} ,d_{2} ) \hfill \\ \, = \beta_{0} + \sum\limits_{i = 1}^{k} {\beta_{i} x_{i} + } \sum\limits_{i = 1}^{k - 1} {\sum\limits_{j = i + 1}^{k} {\beta_{ij} x_{i} x_{j} } + } \sum\limits_{i = 1}^{k} {\beta_{ii} x_{i}^{2} } \hfill \\ = 3.2567 - 0.018234t_{1} + 0.160032t_{2} - 0.0899273d_{1} \hfill \\ - 0.0668366d_{2} - 0.00574433t_{1} t_{2} + 0.0010566t_{1} d_{1} \hfill \\ - 0.000640419t_{2} d_{2} + 0.000802952d_{1} d_{2} { + }0.000515919t_{1}^{2} \hfill \\ - 0.00410596t_{2}^{2} + 0.00053948d_{1}^{2} { + 0}{\text{.000423661}}d_{{2}}^{2} \hfill \\ \end{gathered}$$	0.9602	0.9045	0.0277	0.0834

From the solution accuracy of each response surface form in Table 7, the complete quadratic form performs best in all the four judging criteria. Multiple fitting coefficients *R*^2^ and corrected multiple fitting coefficients *R*_*adj*_^2^ are both close to 1 and the difference between them is only 0.0557, which has superior performance compared with the linear and two-factor interaction models, and shows that the response surface model constructed by applying the complete quadratic model does not contain redundant parameters. Both standard deviation $\sigma$ of 0.0277 and prediction error sum of squares *PRESS* of 0.0834 are at low levels. All four criteria are consistent with the characteristics of good response surface model, so complete quadratic model is used to optimize the gradient foam performance structure.

**Figure 8:**
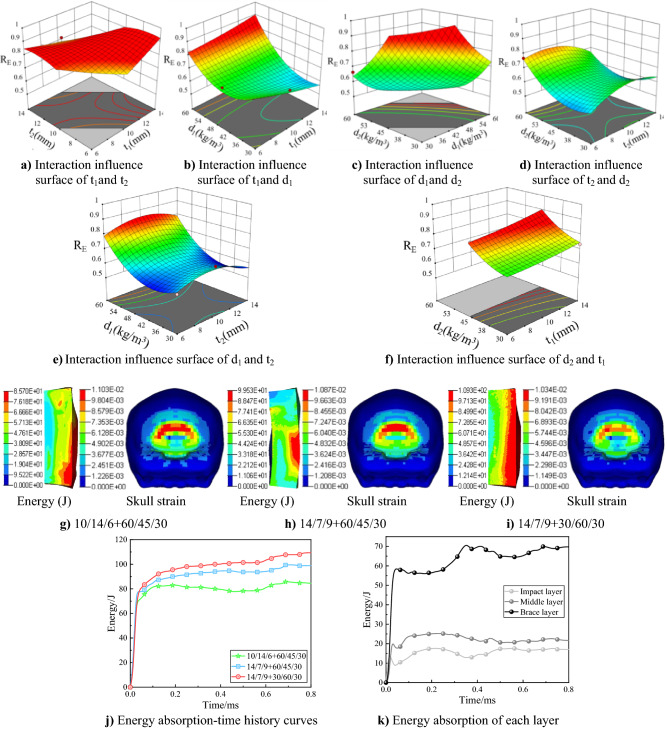
Foam gradient optimization analysis based on RSM.

Figure 8a shows the energy surface plot for interaction of impact layer foam thickness and middle layer foam thickness. There is a certain slope of the whole surface, the slope increases gradually as the impact layer foam thickness taken from low to high while the middle layer foam thickness taken from high to low, which shows that the large impact layer foam thickness and the small middle layer foam thickness have better effect on the improvement of the overall foam structure. Figure 8b shows the interaction effect surface of impact layer foam thickness and density. When the impact layer foam density close to 60 kg/m^3^, the energy absorption ratio is positively correlated with the foam thickness. On contrary, in the interval of lower density, energy absorption ratio is not significantly affected by the thickness, even when the density is 30 kg/m^3^, the energy absorption ratio decreases slightly with the increase of foam thickness. When the density is 60 kg/m^3^ and the thickness is 14 mm, the energy absorption of the foam reaches the maximum. Combined with the impact layer and middle layer foam interaction effect surface (Fig. 8a), it can be seen that the impact layer foam density and thickness in the range of model values are the larger the better. Figure 8c shows the interaction of foam density between the impact layer and middle layer. The energy absorption ratio is significantly influenced by two density variables, and remains low when both the impact layer and middle layer foam densities are in the relatively low region. On the contrary, when the impact layer and middle layer foam density is relatively high and the brace layer foam density is low, the energy absorption ratio has large increase, which consistent with the conclusion obtained in previous bullet high-speed impact experiment and numerical simulation that the energy absorption ratio of brace layer foam is the highest, and the overall energy absorption effect is significant when the relative density of brace layer foam is low. The peak energy absorption ratio occurs approximately at impact layer density of 60 kg/m^3^ and middle layer density of 45 kg/m^3^, the maximum value exceeds the ideal energy absorption value by 90%. Figure 8d shows the interaction between thickness and density of middle layer foam, the surface is saddle-shaped with no obvious positive or negative correlation. Fig. 8e,f also verify the above results, it can be seen from Fig. 8e that when the d_1_ = 60 kg/m^3^, the thickness of middle layer needs to be analyzed in combination with other factors and Fig. 8f shows that there is an optimal solution when t_1_ = 14 mm and d_2_ = 45 kg/m^3^.

In this optimized scheme, the impact layer foam thickness is 14 mm with density of 60 kg/m^3^, the middle layer foam thickness is 7 mm with density of 45 kg/m^3^, the response result is 93.37% of the target optimization value, the model optimal point can be solved as (14, 7.2, 60, 45) from the objective function. According to constraint formula (2) of density and thickness, the optimal design of gradient structure is obtained as t_1_ = 14 mm, t_2_ = 7.2 mm, t_3_ = 8.8 mm, d_1_ = 60 kg/m^3^, d_2_ = 45 kg/m^3^, d_3_ = 30 kg/m^3^, the predicted value R_E_≈0.934, corresponds to the energy absorption value of 96.05 J.

In order to analyze optimization results more objectively, the response surface optimized structural parameters are applied to numerical simulations and compared with the optimal values in the design points before optimization. Since the foam average density in the optimized model is a constant value and there is no convex density gradient with optimal protection performance in the gunshot impact test, the density gradient is replaced by the CVX for simulation on the basis of retaining the optimized thickness gradient and comparative analysis is performed together. Details parameters are shown in Table 8, the skull strain and foam energy absorption under each optimization scheme are shown in Fig. 8g–i. The foam energy absorption-time history curves are shown in Fig. 8j,k shows the foam energy absorption of each layer for the best optimized scheme.

**Table 8:** Design table of optimization scheme.

Parameter	Best solution before optimization	Optimized optimal solution	Optimal thickness + CVX
t_1_	10	14	14
t_2_	14	7	7
t_3_	6	9	9
d_1_	60	60	30
d_2_	45	45	60
d_3_	30	30	30

Figure 8g shows the simulation results of optimal foam combination scheme in the gunshot experiment, the maximum value of cranial strain is 0.01103, the maximum value of energy absorption appears in the brace layer, which is 85.70 J. Figure 8h shows the numerical simulation results of the best solution provided by response surface optimization, the maximum value of cranial strain is 0.01087, the large deformation area is reduced compared with the first optimized solution, the maximum value of foam energy absorption of 99.53 J also appears in brace layer, which is only 3.48 J different from the predicted value of response surface of 96.05 J, indicating that this response surface function reflects the numerical model better, and the energy absorption is improved by 16.14% compared with the first scheme. Figure 8i shows the numerical simulation results of the optimized optimal thickness gradient combined with CVX, with maximum value of 0.01034 for the cranial strain, the maximum energy absorption is almost all over the brace layer, with value of 109.27 J, which is 27.50% and 9.79% higher than the first two solutions respectively.

## Discussion

This study combines rifle bullet impact experiment and finite element simulation to analyze the cranial dynamics response and foam liner energy absorption when different density gradient foam padding is placed behind the bulletproof plate with the rifle bullet high speed impact, then use the response surface method to optimize the thickness and density of each foam padding layer to get the optimal combination.

In the experiment of 5.56 mm rifle impacting bulletproof plate-padding foam-cranial model, the response of various intracranial sites at the moment of impact was recorded using pressure and acceleration sensors. It is evident from the experimental results that the head acceleration values are significantly reduced by using gradient structure, which is consistent with previous researchers' conclusion that the impact resistance and energy absorption capacity of the foam can be improved by using appropriate nonlinear density gradient^19^. Comparing the experimental results for different density gradients, the acceleration and pressure peaks measured in the craniophysical model were the lowest when the graded foam was convex density, this result consistent with the researchers' conclusion that the convex gradient was shown to promote both energy absorption and strength of the graded foam structures in the discretely layered architecture^18^. The finite element model was verified using the images recorded by high-speed photography and the curves measured by the sensors in the experiments, and the skull strain and the energy absorption of the foam were analyzed and discussed to explore the correlation between the gradient foam layers and the overall energy absorption and protection performance. The skull strain was negatively correlated with the energy absorbed by the foam, and the energy absorption of the foam lining was mainly concentrated in the support layer near the head. Combining with the simulation analysis, it can be seen that when foam liner has convex density gradient, the area of cranial strain and large deformation area is relatively small and the energy absorption is higher than other working conditions, the performance is better than that of homogeneous foam, confirming the significance of the study of functional gradient lining materials. The simulation results are consistent with the results obtained from the bullet impact experiments, further illustrating the correctness of the simulation results.

**Figure 9:**
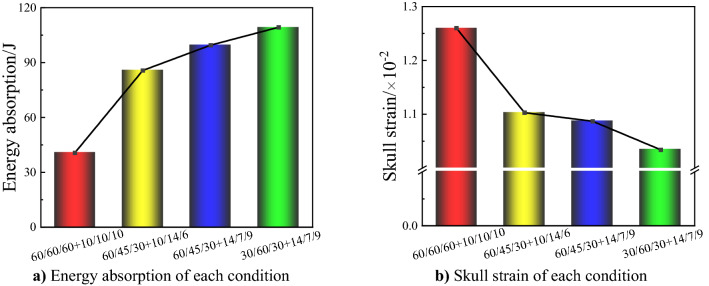
Protection performance of each condition.

In order to further optimize the performance of gradient foam, the influence of different foam layer thickness on the overall structure of energy absorption is considered on the basis of gradient density. For the thickness gradient, the impact layer thickness should be as large as possible within a reasonable range. For the density gradient, the convex density gradient can also reduce the overall weight of the foam while enhancing the protection performance, which has important guiding significance in engineering applications. The relationship between different layer thickness and density is analyzed using the response surface method, and the better thickness ratios are solved after eliminating unrealistic factors. The results optimized by complete quadratic response surface method show that the configuration of large impact layer foam thickness and small middle layer thickness can effectively improve the overall energy absorption effect. Combining the optimal density distribution obtained from the bullet experiment and the optimal thickness distribution obtained from RSM, several different arrangements were loaded into the finite element model, the cranial response and energy absorption of foam liner were simulated and compared. Under the same impact conditions, the optimal foam combination absorbs energy up to 109 J, which is much higher than the homogeneous foam liner of equal thickness with only 40 J energy absorption, and the cranial strain is reduced from 0.0126 to 0.01034 (shown in Fig. 9), the protection performance is greatly improved.

There are still shortcomings in this study, only one foam material has been investigated for protection performance and energy absorption characteristics, lack of comparison of different foam materials, and the gradient design is only carried out for three low densities. Gradient design and impact experiments of other foams will be required in the future to obtain the best solution for protection performance.

## Conclusion

1. The effect of different padding foams on head protection under bullet impact can be analyzed by the energy absorption and the severity of head injury.
2. The liner foam designed for impact brace layer of lower density and thicker thickness, and the middle layer of higher density and thinner thickness can effectively increase the energy absorption.
3. Combining the experimental results and the RSM results, the optimal solutions are 14 mm and 30 kg/m^3^ for the impact layer, 7 mm and 60 kg/m^3^ for the middle layer, 9 mm and 30 kg/m^3^ for the brace layer.
4. The energy absorption of the optimized solution increased by 169% compared to the pre-optimized solution, corresponding to 22% reduction of skull strain, which reduces the damage caused by bullet impact.

## Data Availability

The datasets used and analyzed during the current study available from the corresponding author on reasonable request.
